# Increasing Mechanical Strength of Gelatin Hydrogels by Divalent Metal Ion Removal

**DOI:** 10.1038/srep04706

**Published:** 2014-04-16

**Authors:** Qi Xing, Keegan Yates, Caleb Vogt, Zichen Qian, Megan C. Frost, Feng Zhao

**Affiliations:** 1 Stem Cell and Tissue Engineering Lab, Department of Biomedical Engineering, Michigan Technological University, 1400 Townsend Drive, Houghton, MI 49931; 2 Polymer and Biomaterial Lab, Department of Biomedical Engineering, Michigan Technological University, 1400 Townsend Drive, Houghton, MI 49931

## Abstract

The usage of gelatin hydrogel is limited due to its instability and poor mechanical properties, especially under physiological conditions. Divalent metal ions present in gelatin such as Ca^2+^ and Fe^2+^ play important roles in the gelatin molecule interactions. The objective of this study was to determine the impact of divalent ion removal on the stability and mechanical properties of gelatin gels with and without chemical crosslinking. The gelatin solution was purified by Chelex resin to replace divalent metal ions with sodium ions. The gel was then chemically crosslinked by 1-ethyl-3-(3-dimethylaminopropyl) carbodiimide (EDC). Results showed that the removal of divalent metal ions significantly impacted the formation of the gelatin network. The purified gelatin hydrogels had less interactions between gelatin molecules and form larger-pore network which enabled EDC to penetrate and crosslink the gel more efficiently. The crosslinked purified gels showed small swelling ratio, higher crosslinking density and dramatically increased storage and loss moduli. The removal of divalent ions is a simple yet effective method that can significantly improve the stability and strength of gelatin hydrogels. The *in vitro* cell culture demonstrated that the purified gelatin maintained its ability to support cell attachment and spreading.

Gelatin is a mixture of proteins obtained by acid or alkaline hydrolysis of collagen. Gelatin has excellent biocompatibility, biodegradability, non-immunogenicity, and great capacity for modification at the level of amino acids^1^,^2^,^3^,^4^,^5^. In addition, gelatin is abundantly available from nature, easily processed into various shapes and forms, and inexpensive^1^,^2^,^3^. Due to its superior properties, gelatin has been extensively employed as a biomaterial for both hard and soft-tissue engineering, drug delivery, and biological glues^2^,^3^,^6^.

A gelatin hydrogel can be formed by physical crosslinking in water above a certain concentration (around 2% w/v) and below 30 ~ 35°C^7^,^8^,^9^,^10^. During the process, gelatin molecules aggregate and undergo a conformational change from a random coil to a triple helix^7^,^8^,^9^,^10^. At the same time, intermolecular hydrogen bonds form between large fractions of gelatin chains. However, the non-covalent associations are easily broken at temperatures higher than 30 ~ 35°C, thereby destroying the physical network. The gelatin hydrogels thus have low shape stability, poor mechanical strength, and low elasticity^11^, which significantly limit their biomedical applications at physiological temperatures, 37°C. To increase its stability and mechanical properties, the gelatin gel can be covalently crosslinked by small chemicals such as carbodiimides, formaldehyde, and glutaraldehyde, which can couple the carboxyl groups with amino groups, forming stable amide bonds^12^,^13^,^14^,^15^,^16^. The crosslinked gelatin can form an intricate high molecular weight network that is capable of swelling. Nevertheless, this processing approach is easy to generate domains with non-uniform crosslinking^17^. The effects of introducing other polymers into the gelatin have been studied in an attempt to overcome the restrictions on the application of gelatin. For example, oxidized cellulose nanowhiskers have been used to crosslink gelatin matrix to increase its mechanical properties and resistance towards thermal degradation^11^. Poly(lactic-co-glycolic acid) microspheres were incorporated into gelatin based hydrogel to improve the tensile strength^18^. Gelatin-PHEMA hydrogel shows enhanced elastic modulus when the amount of PHEMA is increased^19^. However, the introduction of additional polymers may produce undesirable side effects, such as decreasing the superb non-immunogenic property of gelatin.

As a denatured product of collagen, gelatin contains many divalent metal ions such as calcium, copper, iron and zinc that can form ionic bonds with the carboxylic acid groups on the gelatin polypeptides, influencing the organization of the gelatin network. However, there is little information on the impact of divalent ions on the stability and mechanical properties of gelatin hydrogels. It is anticipated that removal of metal ions in gelatin will free the carboxylic acid groups in the polypeptide molecules, thereby strengthening the electrostatic interactions between the carboxylic acid groups and amine groups within and between the polypeptide molecules, and also improving the crosslinking density upon chemical crosslinking, eventually significantly improving the mechanical strength and stability of the gelatin hydrogel. The objective of this study was to introduce a novel yet simple approach to increase the mechanical properties and stability of gelatin gels by removing divalent metal ions using Chelex resin. Here, the gelatin network formation, gel swelling, gel degradation, water contact angle, and mechanical strength before and after the divalent ion removal were investigated. The cell attachment was also observed by culturing human mesenchymal stem cells (hMSCs), which are easily obtained adult stem cells that show wide and significant use in biomedical applications^20^,^21^, on top of the gelatin hydrogel. Results showed that the removal of divalent metal ions could significantly enhance the storage and loss moduli as well as the stability of the gelatin hydrogels after chemical crosslinking without affecting the cell attachment on the gelatin hydrogel.

## Results

### Measurement of divalent ion concentration

**Figure 1:**
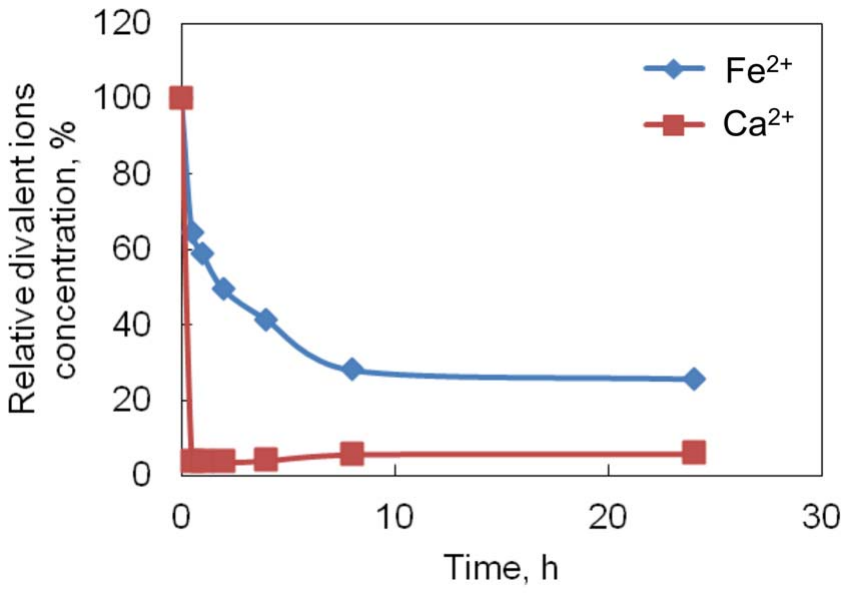
The change of Ca^2+^ and Fe^2+^ concentration with respect to time during the purification process. The ion concentration was expressed as the percentage of initial concentration.

The divalent ions concentration in gelatin was measured by Inductively Coupled Plasma – Optical Emission Spectroscopy (ICP-OES). The results in Figure 1 demonstrated that the amount of Fe^2+^ and Ca^2+^ in gelatin significantly decreased after the 24 h purification process (divalent ions removal process). Around 75% Fe^2+^ and 95% Ca^2+^ were removed from the gelatin. The dynamic measurement of ion concentration showed that both Fe^2+^ and Ca^2+^ content sharply decreased in the first 30 min. Then Fe^2+^ gradually decreased from 64% to 25% of the original concentration after 24 h, while Ca^2+^ maintained around 5% of the original concentration throughout the following 24 h. The end point concentrations of Fe^2+^ and Ca^2+^ in the gelatin gels were 0.03 mM and 0.1 mM, respectively.

### Swelling behavior

**Figure 2:**
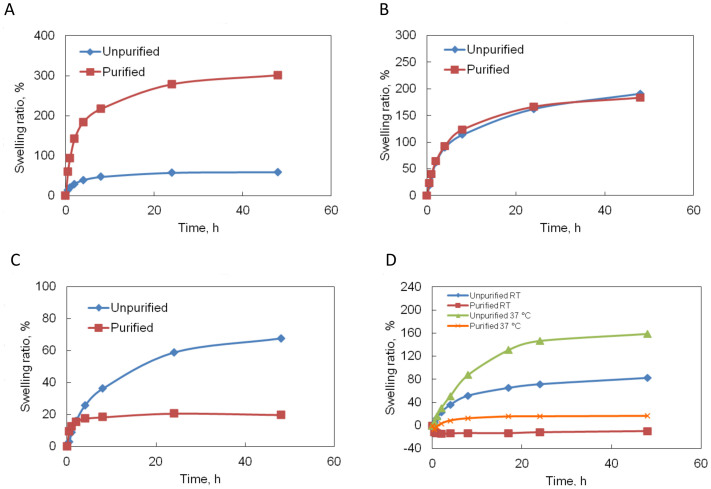
Effect of purification, solvent and EDC crosslinking on swelling ratio of gelatin hydrogels. (A). Swelling of uncrosslinked hydrogel in DIH_2_O at room temperature. (B). Swelling of uncrosslinked hydrogel in PBS at room temperature. (C). Swelling of crosslinked hydrogel in DIH_2_O at room temperature. (D). Swelling of crosslinked hydrogel in PBS at room temperature (RT) and 37°C.

Swelling ratio has been widely used as a simple method to characterize water absorption and stability of biomaterials^22^. The swelling behavior of uncrosslinked and EDC-crosslinked gelatin hydrogel samples in deionized water (DIH_2_O) and PBS as a function of time was depicted in Figure 2. It was found that most of the gels attained equilibrium swelling by 48 h. The uncrosslinked gelatin hydrogel samples showed dramatically different swelling behavior in DIH_2_O and PBS at room temperature (see Figure 2 A and B). After 48 h swelling in DIH_2_O, the swelling ratio of purified gelatin hydrogel reached 301 ± 15%, whereas the swelling ratio of unpurified gelatin hydrogel was only 59 ± 4.4%. However, these two samples had similar swelling ratios at all time points when soaked in PBS. The equilibrium swelling ratios were 184 ± 5.7% and 191 ± 4.4% for purified and unpurified samples, respectively.

After chemical crosslinking, the swelling ratio decreased for all the samples as a result of the formation of a rigid network. The unpurified gelatin gels consistently demonstrated higher swelling capacity than purified gelatin gels in both DIH_2_O and PBS (Figure 2 C and D). At room temperature in DIH_2_O, the swelling ratio of unpurified gelatin gels was 2.4 fold higher than the purified gelatin gels after 48 h. At room temperature in PBS, the swelling ratio of unpurified gelatin gels was 83 ± 7.1% whereas the swelling ratio of purified gelatin gels was only −9.6 ± 1.1%. It could be explained that PBS has much higher ionic strength (0.16 M) than the water (close to 0 M) contained in purified gelatin gels, therefore the gel lost water due to the osmotic pressure. This effect surpassed the water absorption ability of the gels, which made the sample weight decrease. Under physiological relevant conditions (37°C, in PBS), the swelling ratio of purified gelatin gels reached 17 ± 5.6%, which is 8.4 times (*p* < 0.05) less than the unpurified samples 159 ± 1.4%.

### Crosslinking density

Unpurified and purified gelatin gels crosslinked with EDC were allowed to swell in PBS at 37°C for three days to reach swelling equilibrium. The crosslinking density was calculated using the modified Flory swelling equation as reported in a previous publication^23^. Table 1 lists the swelling measurements and related parameters as an independent estimation of the crosslinking density. The calculation was based on the following given values: molar volume of the solvent *V*_1_ (18 mL/mol), the Flory-Huggins polymer-solvent interaction parameter *χ*_1_ (0.495)^23^, solvent density *ρ*_1_ (1.00 g/cm^3^), and polymer density *ρ*_2_ (1.35 g/cm^3^). The crosslinking density, expressed as the crosslink network chain per gram, in EDC-crosslinked unpurified gelatin gel is significantly lower than that in EDC-crosslinked purified gelatin gel as demonstrated in Table 1.

### FT-IR characterization

**Figure 3:**
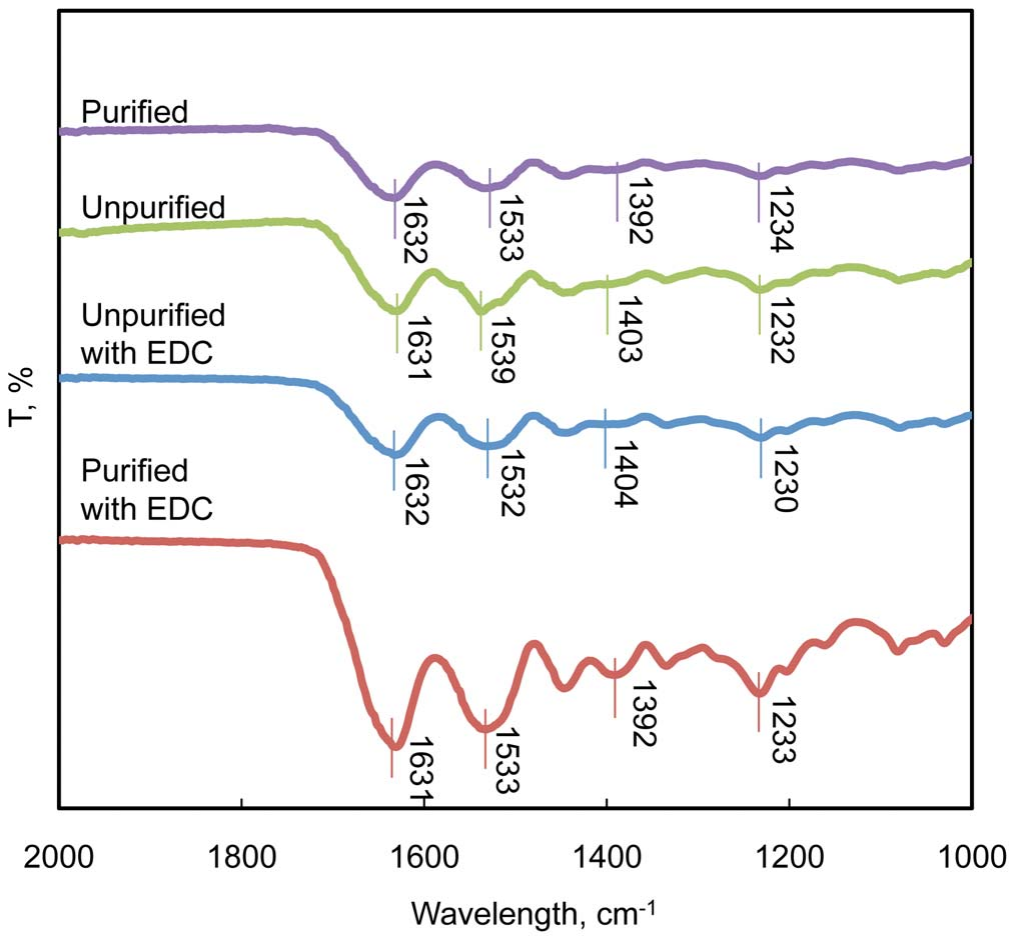
FTIR spectra for uncrosslinked and EDC-crosslinked purified and unpurified gelatin hydrogels.

The FT-IR spectra of purified and unpurified hydrogels with or without EDC crosslinking is shown in Figure 3. The characteristic absorption peak around 1631 cm^−1^ was typical for a stretch of C = O. The peak around 1532 cm^−1^ corresponds for N-H deformation. The 1404 cm^−1^ peak was for C-N stretching and 1230 cm^−1^ peak for N-H bending. By comparing the four samples, none of the characteristic peaks shifted significantly, indicating that the purification process did not change the chemical structure of gelatin molecules. The C-N peak intensity at 1404 cm^−1^ of EDC-crosslinked purified gelatin was higher than all the other samples, which might indicate that the EDC-crosslinked purified gelatin hydrogels contained higher amount of amide bonds due to the higher EDC crosslinking density.

### Shape stability and morphology

**Figure 4:**
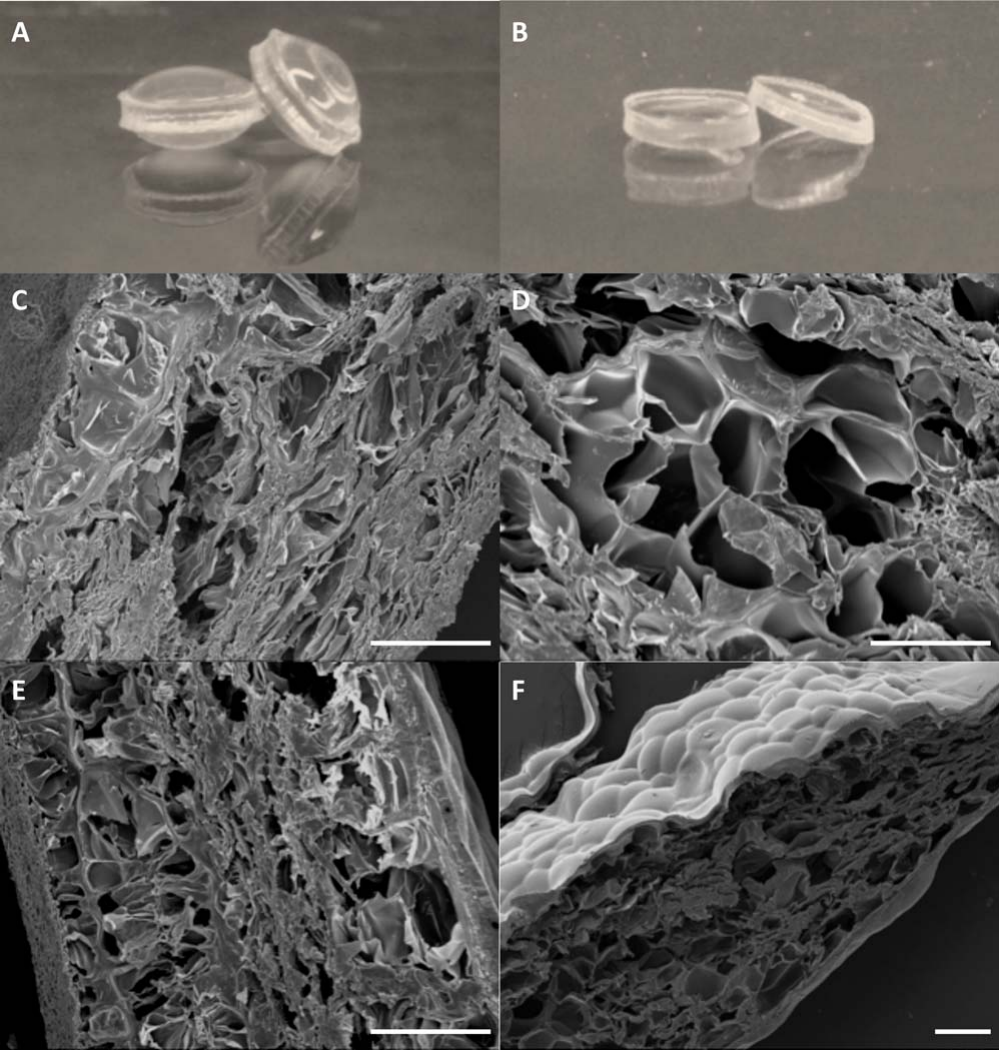
Morphologies of unpurified and purified hydrogels. Gross appearance of crosslinked unpurified (A) and purified (B) gels after 48 h swelling in PBS at 37 °C. SEM images of transverse cross sections of unpurified (C) and purified (D) gels before EDC crosslinking, and unpurified (E) and purified (F) gels after EDC crosslinking. Scale bar: 150 μm.

The shape and size of the purified gelatin samples after the EDC crosslinking did not change significantly during the whole swelling process at 37°C (Figure 4 B). On the contrary, the unpurified samples after the EDC crosslinking absorbed a large amount of water, especially in the core of the samples. Therefore, the unpurified samples did not swell evenly, but formed a bulge in the middle part (Figure 4 A). To investigate the underlying mechanism, the morphologies of unpurified and purified gelatin hydrogels were observed with SEM. Both unpurified and purified gelatin hydrogels formed a porous and interconnected network before EDC crosslinking (Figure 4 C, D). It was obvious that the purified gelatin samples exhibited much larger pores and more uniform structures than the unpurified gelatin samples. After EDC crosslinking, the micro-scale morphologies did not change significantly for either sample. The EDC-crosslinked unpurified gelatin samples formed a compact gel shell at the edge as observed in Figure 4 E. The EDC-crosslinked purified samples also formed a shell, but much thinner and more penetrable due to the relaxed conformation of gelatin molecules (Figure 4 F).

### In vitro degradation

**Figure 5:**
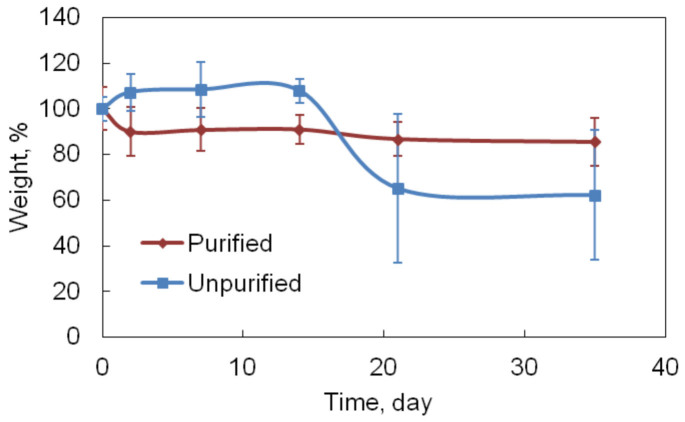
The *in vitro* hydrolysis degradation behavior of purified and unpurified gelatin hydrogels under pH 7.4 and 37°C.

The *in vitro* degradation of the gelatin hydrogel in PBS at 37°C is another important property that determines the stability of the samples under physiological relevant conditions. Figure 5 depicted the *in vitro* degradation results of EDC-crosslinked unpurified and purified gelatin gels in PBS at 37°C. The purified gelatin gels lost about 10% of the dry weight in the first two days and maintained a similar dry weight in the following 5 weeks. The unpurified gelatin gels absorbed much more PBS and the salt remained in the gel after freeze-drying, thus the dry weight increased in the first 2 weeks. At later stage, some of the unpurified gels were broken and the liquid-like content flowed out, causing a dramatically decreased dry weight. After 5 weeks incubation in PBS, the purified hydrogel preserved 86 ± 10 wt% of the original dry weight, whereas its unpurified counterpart contained only 62 ± 28 wt% dry weight of the original mass. These results demonstrated that the EDC-crosslinked purified gelatin gel had excellent stability in physiological relevant conditions.

### Mechanical properties

**Figure 6:**
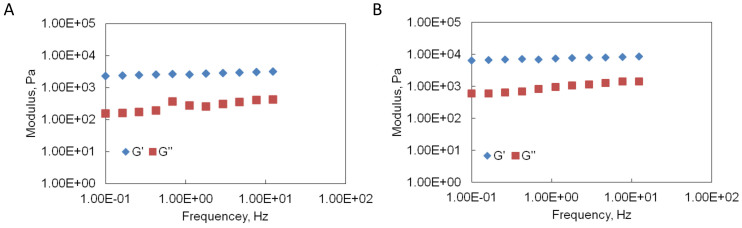
Comparison of storage G′ and loss G″ moduli of freshly prepared gelatin hydrogels before EDC crosslinking. (A). Purified gelatin gels. (B). Unpurified gelatin gels.

The frequency dependence of the storage modulus (G′) and loss modulus (G″) for uncrosslinked gelatin hydrogels was depicted in Figure 6. For all the hydrogel samples, the G′ was almost one order of magnitude higher than G″ over the frequency sweep (0.1–1.2 Hz) indicating predominant elastic response. Before EDC crosslinking, the unpurified gelatin gels had better mechanical strength than purified gelatin gels. The average storage modulus and loss modulus of unpurified gelatin gels were 2.83 ± 0.10 fold and 3.80 ± 0.43 fold higher than purified gelatin gels. After EDC crosslinking, the average storage modulus and loss modulus of purified gelatin gel dramatically increased 16 ± 4.4 fold and 100 ± 14 fold; while the average storage modulus and loss modulus of unpurified gelatin gels increased 2.1 ± 0.65 fold and 18 ± 2.0 fold. In the tensile strength test, the ultimate tensile stress that the EDC-crosslinked unpurified gelatin gels could withstand was 107 ± 25 KPa. For the EDC-crosslinked purified gelatin gels the ultimate tensile stress was 196 ± 17 KPa, which was 83% (*p* < 0.05) higher. The ultimate strain of EDC-crosslinked purified gelatin gels was 2.8 ± 0.46 fold of EDC-crosslinked unpurified gelatin gels. The tensile strength test could not be performed on gelatin gels without EDC crosslinking due to the extremely weak mechanical strength.

**Figure 7:**
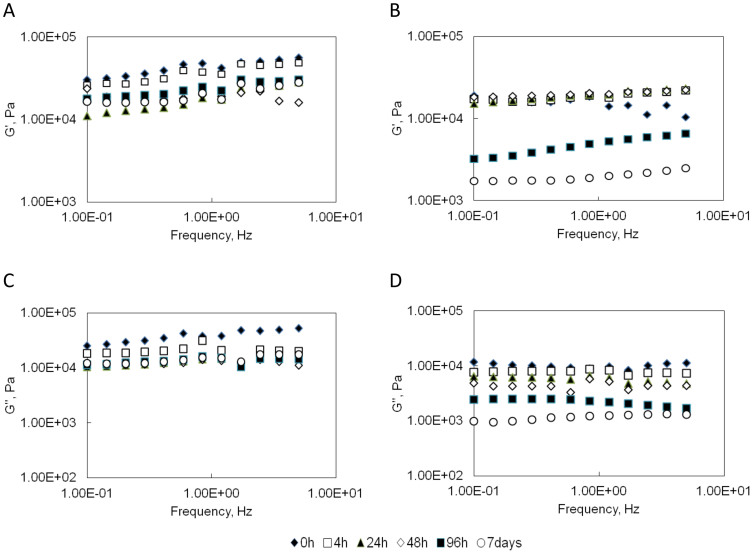
Frequency sweeps of EDC-crosslinked gelatin hydrogels swollen at different time points. (A). Storage modulus (G′) of purified gelatin gels. (B). Storage modulus (G′) of unpurified gelatin gels. (C). Loss modulus (G″) of purified gelatin gels. (D). Loss modulus (G″) of unpurified gelatin gels.

Frequency sweeps were used to determine the mechanical properties of the EDC-crosslinked gelatin hydrogels at the different swelling time intervals, as shown in Figure 7. In general, the mechanical strength of both purified and unpurified gelatin gels continuously decreased when soaked in PBS at 37°C. Both G′ and G″ of purified gelatin samples gradually decreased up to 24 h swelling time. After this time point, changes of G′ and G″ were not significant. Interestingly, the drops of G′ and G″ of unpurified gelatin samples were more prominent in the later stage day 4 and day 7.

The changes of G′ and G″ on the swelling time of the EDC-crosslinked gelatin hydrogels at the frequency of 0.42 s^−1^ were recorded in Table 2. At the initial time point, the average G′ and G″ of purified gelatin gel is 2.4 ± 0.8 fold and 3.5 ± 0.2 fold higher than unpurified gelatin gel. After 7 days of soaking in PBS, the average G′and G″of purified gelatin gel decreased 58 ± 3.1% and 63 ± 7.9%; the average G′ and G″ of unpurified gelatin gel decreased 89 ± 3.2% and 88 ± 3.8%. The unpurified gels lost their mechanical strength much more quickly than the purified gels. At the final time point, the average storage modulus and loss modulus of the purified gelatin gel were 8.2 ± 0.3 fold and 10.3 ± 0.5 fold higher, respectively, than the unpurified gelatin gel.

### Effects of Ca^2+^ content on mechanical properties

**Figure 8:**
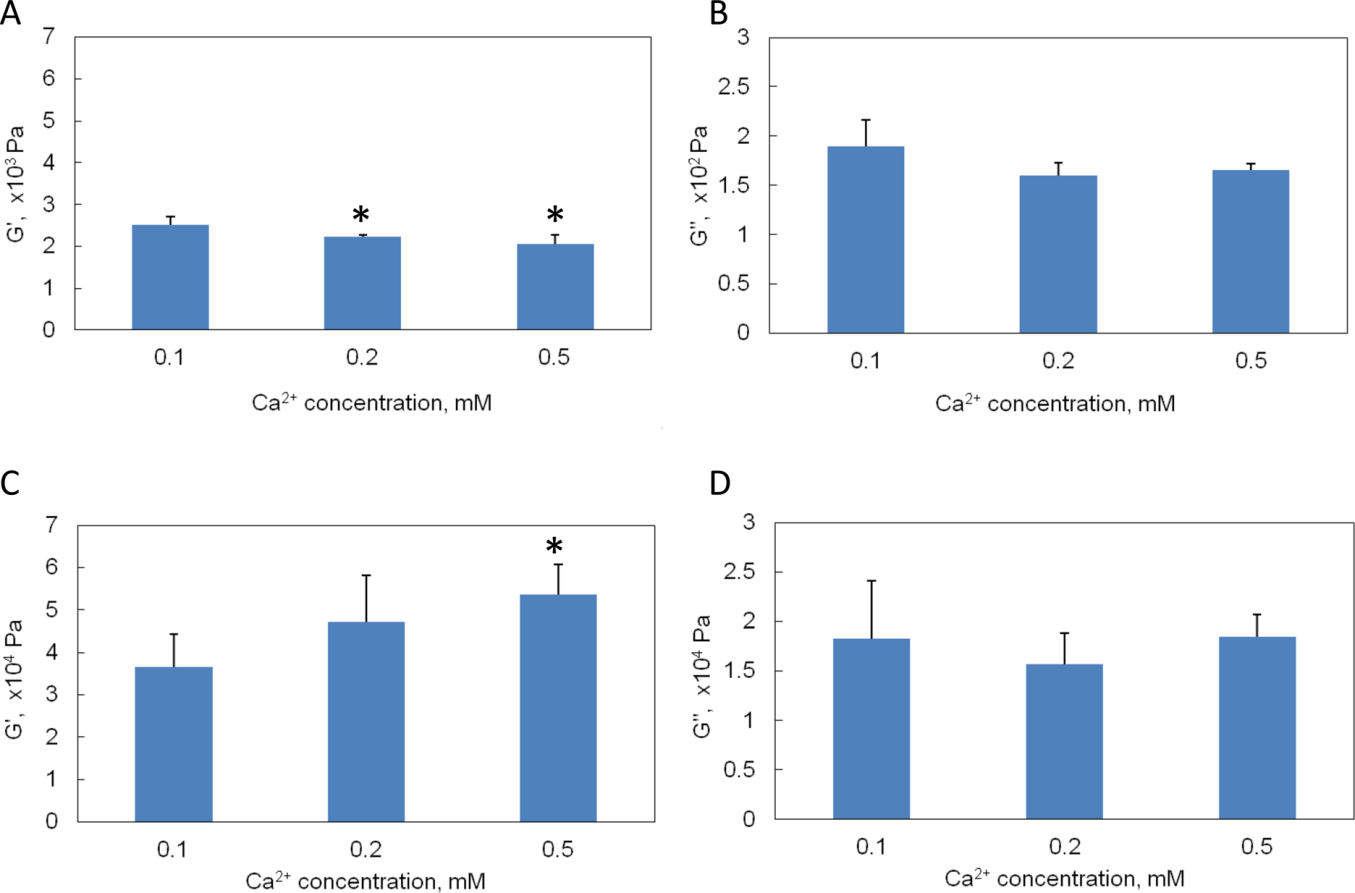
Effects of exogenously introduced Ca^2+^ ions in the purified gelatin solution on mechanical properties of gelatin hydrogels. (A). Storage modulus (G′) of uncrosslinked gelatin gels. (B). Loss modulus (G″) of uncrosslinked gelatin gels. (C). Storage modulus (G′) of crosslinked gelatin gels. (D). Loss modulus (G″) of crosslinked gelatin gels. * *p* < 0.05 compared with samples containing 0.1 mM Ca^2+^.

The Ca^2+^ content in gelatin was quickly reduced to around 5% in 30 minutes by Chelex resin. In order to correlate the effect of various Ca^2+^ amount on mechanical properties of gelatin gels with and without chemical crosslinking, gelatin hydrogels with different amount of Ca^2+^ were fabricated by adding CaCl_2_ into the purified gelatin solution. The Ca^2+^ concentration was adjusted to 0.1, 0.2 and 0.5 mM, which correspond to 5%, 10% and 25% of Ca^2+^ content in unpurified gelatin gel. The storage (G′) and loss (G″) moduli of uncrosslinked and EDC-crosslinked hydrogels with respect to the Ca^2+^ content were shown in Figure 8. For uncrosslinked hydrogels, when Ca^2+^ concentration increased from 0.1 mM to 0.2 and 0.5 mM, the average G′ decreased around 11 ± 5.4% (*p* < 0.05) and 19 ± 2.5% (*p* < 0.05), respectively. However, the average G″ did not change significantly. For EDC-crosslinked hydrogels, when Ca^2+^ concentration increased from 0.1 mM to 0.2 and 0.5 mM, the average G′ increased 29 ± 2.8% (*p* > 0.05) and 48 ± 12% (*p* < 0.05), respectively. And G″ were comparable at all three Ca^2+^ concentrations.

### Cell attachment and morphology

**Figure 9:**
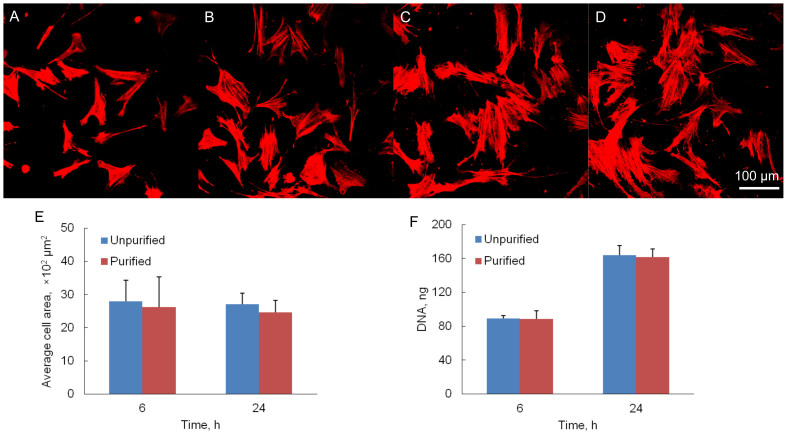
*In vitro* hMSC culture on gelatin hydrogels. F-actin staining of cells on (A). Purified gel at 6 h; (B). Unpurified gel at 6 h; (C). Purified gel at 24 h; (D). Unpurified gel at 24 h. (E). Average cellular area covered by each cell; (F). Cell attachment at 6 and 24 h after seeding. Error bars = means ± SD (n = 3).

In order to investigate the ability of EDC-crosslinked purified and unpurified gelatin hydrogels to support cell attachment, the hMSCs were seeded on both hydrogel. The confocal images in Figure 9 (A–D) showed that hMSCs attached and spread well on both the gelatin hydrogel surfaces after 6 and 24 h cell seeding. The average area covered by each cell at 6 and 24 h for these two samples did not show significant difference (*p* > 0.05), as illustrated in Figure 9 E. Cells exhibited normal morphology on both purified and unpurified gelatin hydrogel surfaces. There was no significant difference (*p* > 0.05) in the total DNA amount from attached cells for both purified and unpurified gels at the two time points, as shown in Figure 9 F. At 6 h, the average cell attachment ratios were 36 ± 3.8% and 36 ± 1.5% for purified and unpurified samples, respectively. At 24 h, the average cell attachment ratios increased to 65 ± 4.0% and 66 ± 4.6% for purified and unpurified samples, respectively.

## Discussion

**Figure 10:**
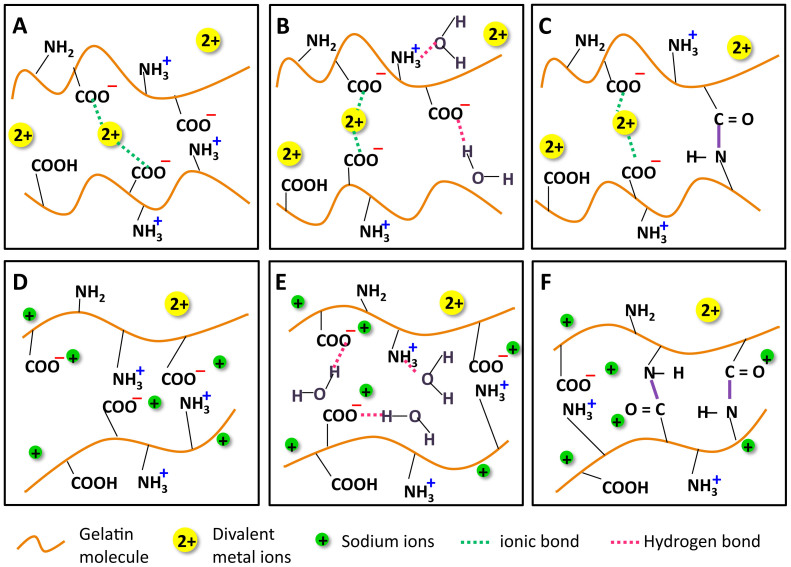
Schematic representing the possible interactions between ions and gelatin molecules, formation of hydrogen bonds during swelling and amide bonds during chemical crosslinking in unpurified (A), (B), (C) and purified gels (D), (E), (F).

Gelatin contains trace amount of divalent metal ions such as Cu^2+^, Ca^2+^, and Fe^2+^, which play important roles in the gelatin gel network formation^24^,^25^,^26^. These ions form ionic bonds with carboxylic acid groups on the gelatin molecule chains, as shown in Figure 10 A. During the gelation process, the gelatin peptides aggregate and physically crosslink with each other to form the gel. The presence of divalent metal ions could accelerate the protein aggregation^27^. Some negatively charged carboxylic acid groups also attract positively charged amine groups through electrostatic interactions (Figure 10 A). The gelatin polymer network is highly hydrophilic, which absorbs water through hydrogen bonds formed between water molecules and carboxylic acid and amino groups (Figure 10 B). During the EDC crosslinking process, the carboxylic acid groups are covalently linked with amino groups via the formed amide bonds (Figure 10 C).

The Chelex resin significantly removed divalent metal ions such as Ca^2+^ and Fe^2+^ after purification process. The divalent metal ions in gelatin were replaced with equivalent amount of Na^+^ originally in the resin, as shown in Figure 10 D. The energy dispersive spectrum (EDS) examination of lyophilized hydrogels confirmed that the amount of Na^+^ greatly increased in purified samples (Supplemental Figure 1). The Na^+^ had a weaker electrostatic interaction with negatively charged -COO^−^ than the divalent metal ions, leading to more free carboxylic acid groups, which increased the hydrophilicity of the gel. This conclusion was proved by the contact angle of the unpurified gelatin gel (129.2 ± 4.6°) and the purified gelatin gel (69.5 ± 6.7°). Meanwhile, higher amount of water was absorbed in the polymer network because more hydrogen bonds were formed between the freed carboxylic groups and water molecule (Figure 10 E -). The deficiency of divalent ions in gelatin resulted in less aggregated protein chains and consequently a looser structure. So the EDC solution can easily penetrate into the hydrogel and crosslink more carboxylic acid groups (Figure 10 F) and the amino groups compared with the unpurified samples (Figure 10 C). This has been confirmed by the estimation of degree of crosslinking in Table 1. Chelex resin also slightly changed the pH of the solution. After purification, the pH of gelatin solution increased from 6.5 to 7.0. Since the isoelectric point of gelatin is between 4.7–5.2, the carboxylic acid groups are totally deprotonated in both unpurified and purified gelatin solutions. Therefore, the –COO^−^ groups carried the same total charge and did not significantly affect the subsequent EDC crosslinking efficiency.

The swelling behavior of uncrosslinked gelatin hydrogels in DIH_2_O is affected by the hydrophilic groups present in the polymer chains. After purification, the stronger ionic bonds between –COO^−^ groups and divalent metal ions were broken and replaced by a weaker ionic interaction between –COO^−^ and Na^+^ ions (Figure 10 D). The easier disassociation of –COO^−^Na^+^ resulted in abundant availability of hydrophilic groups –COO^−^, which made the purified gelatin gels form more hydrogen bonds, and thereby absorb much more water than the unpurified gelatin gels (Figure 2 A). However, when the solvent was changed to PBS, which contained a high concentration of Na^+^ and K^+^, the absorption of solvent is strongly influenced by Donnan effect or the mobile ion (Na^+^) concentration difference between the ions inside the gel and the ions in PBS solution. Since purified gelatin gels contain a greater amount of Na^+^, the mobile ion concentration difference is relatively small. Thus, the purified gelatin samples absorbed less solvent in PBS than in DIH_2_O. For unpurified gelatin gels, the mobile ion concentration difference is relatively higher. Thus, the swelling ratio increased for unpurified gelatin samples when the solvent was switched from DIH_2_O to PBS (Figure 2 A, B).

After EDC crosslinking, the equilibrium swelling ratio decreased for all gelatin samples. Similar behavior was also observed in other gelatin gel systems crosslinked with glutaraldehyde, periodated alginate and cellulose nanowhiskers^11^,^28^,^29^. According to the theory of equilibrium swelling, the swelling ratio of the gels is related to their effective crosslinking density. Swelling measurement is generally accepted as a proper method to estimate the crosslinking density of a hydrogel^30^. The swelling-determined crosslinking density offers an inexact, but useful comparison between two different gelatin gel systems. As mentioned before, the deficiency of divalent metal ions in purified gelatin can free the carboxylic acid groups in polypeptide molecules, improving the crosslinking density upon chemical crosslinking. The independent calculation of crosslinking densities, as shown in Table 1, also confirmed that purified samples had significantly higher EDC crosslinking density. Therefore, the purified gelatin gel network had much lower swelling ratio than its unpurified counterpart after EDC crosslinking (Figure 2 C, D) in both DIH_2_O and PBS.

The different physical interactions between and inside the gelatin molecules resulted in different network formation. Before EDC crosslinking, the purified gelatin samples had weaker physical “cross-links” than unpurified samples due to the loss of divalent ions. Thus, purified samples had relatively open network structure with larger pores (Figure 4 D) that facilitated the diffusion of EDC crosslinking solution into the inner region of hydrogels and the formation of a relatively uniform structure throughout the gels. It was relatively difficult for the EDC solution to penetrate into the tight structure of unpurified gelatin gels. Thus, it is plausible that the outer region had higher crosslinking density than the inner region. The high concentration of gelatin (15 wt%) resulted in strong physical interactions between gelatin chains and the formation of gels with good stiffness. Thus, the subsequent chemical crosslinking did not significantly change the microstructure (Figure 4 E, F). Because the inner part of the crosslinked unpurified samples had lower crosslinking density than the outer part, the inner part absorbed much more water. Some samples swollen at 37°C even broke apart and the gelatin inside the shell became liquid-like components.

The mechanical properties of the different gelatin gels were also highly dependent on the gelatin molecule interactions and the degree of chemcial crosslinking. Due to the physical interactions between the gelatin molecules and divalent metal ions, the mechanical strength of unpurified gelatin gels was higher than purified gelatin gels before EDC crosslinking (Figure 6). While after EDC crosslinking, the purified gelatin gels became much stronger and more elastic than unpurified gelatin gels because of the higher crosslinking density (Figure 7). During the swelling process, the hydrogels absorbed large amounts of water that increased the mobility of the gel network and decreased the gel stiffness. The change of mechanical strength of EDC-crosslinked gelatin gels was consistent with the swelling behavior and the degradation result. The overall swelling ratio and mass loss of crosslinked purified gelatin hydrogels were lower than the crosslinked unpurified gels. Correspondently, the overall loss in mechanical strength for crosslinked purified gelatin gels was smaller than the crosslinked unpurified gels. A similar phenomenon was observed in sodium alginate/gelatin system where the viscoelatic properties complied with the swelling behavior^31^. However, the crosslinked unpurified gelatin samples had a tight network structure at the outmost region and did not swell as much as the inner region. Although the unpurified gelatin hydrogels absorbed a large amount of solvent in the first 48 h, the surface remained stiff and the G′ value did not change significantly. In the later stage, the surface started to soften and some samples were broken, leading to rapid decrease of G′ and G″. Due to the fact that the water content in purified samples was always lower than the unpurified samples, the glassy behavior was better maintained and high values of storage and loss modulus were recorded.

To further investigate the effect of different concentration of divalent ion on the mechanical properties of gelatin hydrogels before and after crosslinking, Ca^2+^ ions were added back into the purified gelatin solution in the form of electrolyte CaCl_2_. Rheometry testing showed that the storage modulus decreased with higher salt concentration before EDC crosslinking. However there was no significant change found in the loss modulus. It is possible that when Ca^2+^ was added back into purified gelatin solution, the presence of large amount of Na^+^ interfered with the ionic interactions between Ca^2+^ and carboxylic acid groups. Furthermore, the CaCl_2_ acted as a salt to deteriorate the cohesion of triple helix constituents in gelatin molecules^32^, which resulted in a relatively loose structure. After EDC crosslinking, storage modulus increased with higher salt concentration. This phenomenon might be related to the easy penetration of EDC solution in the relatively loose structure.

Human mesenchymal stem cells, as a model cell line^33^,^34^, were employed to detect the influence of ion removal on the cell attachment property of the gelatin hydrogel. Gelatin contains abundant Arg-Gly-Asp (RGD) sequences which are the cell attachment sites recognized by many integrins^35^. The presence of RGD sequences facilitates cell adhesion and spreading. The *in vitro* cell culture results demonstrated that cell attachment ratio and cell morphology were comparable for purified and unpurified gelatin hydrogel. This phenomenon indicates that the ion-removal and the subequent EDC crosslinking process did not cause a negative effect on cell attachment and spreading.

In this work, the impact of divalent ions removal on the stability and mechanical strength of gelatin hydrogel was presented. The morphologies, swelling behavior, *in vitro* degradation and mechanical strength changes of gelatin hydrogels were investigated under physiologically relevant conditions. Results demonstrated that the absence of divalent metal ions remarkably improved the uniformity and effectiveness of the crosslinker within the gelatin hydrogel upon chemical crosslinking. The EDC-crosslinked purified gelatin hydrogels had higher EDC crosslinking density, which led to a lower swelling ratio, a more stable structure, and a significantly higher mechanical strength when compared to unpurified samples. The divalent ion removal can significantly enhance the stability and mechanical properties of gelatin without introducing extra filler or polymer substances. Furthermore, the purified gelatin maintained its biocompatibility to support cell attachment and spreading. Therefore, the purified gelatin hydrogels may be a suitable alternative to unpurified gelatin for use in tissue engineering applications that require higher stiffness and water resistance. Our finding also show the potential in controlling the hydrogel structure and stability via mediating divalent ion concentrations in the system.

## Methods

### Materials

Gelatin (type B) from bovine skin and N-(3-Dimethylaminopropyl)-N′-ethylcarbodiimide hydrochloride (EDC) were purchased from Sigma-Aldrich (St. Louis, MO). Chelex 100® resin was purchased from Bio-Rad (Hercules, CA).

### Preparation of gelatin gels

The unpurified gelatin solution was prepared by dissolving gelatin powder in deionized water (DIH_2_O) at 60°C under continuous stirring until the solution became homogenous. The purified gelatin solution was obtained as previously described^36^. Briefly, Chelex 100® resin was added into the unpurified gelatin solution (5 g/100 ml) and continuously stirring for 24 hours at 60°C. The supernatant was collected at different time points to test divalent ions. The supernatant collected at 24 h was designated as purified gelatin solution. Different amount of CaCl_2_ was added back into the purified gelatin solution in order to test the influence of the Ca^2+^ concentration on the mechanical strength of resulted gels. The prepared gelatin solutions were poured into plastic Petri dishes and transferred to a refrigerator. After 24 hours cooling at 4°C, the gels were cut using circular molds and chemically crosslinked for 24 hours at room temperature with EDC (5 mM) dissolved in a mixture of acetone/water (90/10 v/v). The pH value of the gelatin solutions was measured by Accumet™ multi-parameter pH meter (Fisher Scientific, Pittsburgh, PA).

### Ion content measurement

The ion content of the gelatin was determined with Optima 7000DV ICP-OES (Perkin Elmer, MA). Briefly, the gelatin solution was allowed to form gels and freeze-dried for 2 days and burned at 500°C for one hour. The obtained ash was dissolved in 1 M HCl and filtered for ICP test. Three repeats were performed for each experimental condition.

### Swelling behavior

The gelatin hydrogels were immersed in deionized water and phosphate buffered saline (PBS) (pH 7.2) at room temperature or 37°C, respectively. The samples were taken out from PBS at selected time intervals, wiped with wet tissue paper to remove surface droplets, weighed, and placed back in PBS. The swelling ratio *Q_m_* was calculated using the following equation: 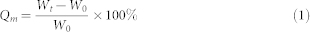
where *Wt* was the weight of the swollen sample at certain time point, and *W*_0_ was the initial weight of the sample.

### Estimation of crosslinking density

The crosslinking density of the gelatin hydrogel was estimated from the swelling measurement using the following equation as reported earlier^23^: 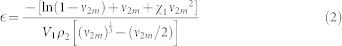
where 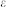 is the crosslinking density,*V*_1_ is the molar volume of the solvent, *ν*_2*m*_ is the volume fraction of polymer in the swollen network at equilibrium, *χ*_1_ is the Flory-Huggins polymer-solvent interaction parameter. *ν*_2*m*_ is calculated from the mass swelling ratio *Q_m_*, 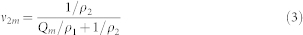
where *ρ*_1_ is the solvent density and *ρ*_2_ is the polymer density.

### Contact angle measurement

The contact angle of unpurified and purified gelatin hydrogels were measured using G10 contact angle measurement system (Krüss, Germany). DIH_2_O was used to form pendant droplets.

### Tensile strength test

Stress-strain analysis of gelatin gels was performed by making uniaxial measurements using an Instron tester 8872 (Instron, Norwood, MA) equipped with a 2 lb load cell. Gelatin gels were cut into bone-shape specimens with a cross-section area of 3.5 × 1.5 mm. They were then clamped vertically, with a gauge length of 35 mm and tested at a constant rate of 0.17 mm/s. All samples were stretched until failure. Stress was calculated by dividing the force generated during extension by the initial cross-sectional area.

### Rheological measurements

All rheological experiments were conducted using an Bohlin CVOR rheometer (Malvern Instruments, UK). The parallel plate diameter used was 15 mm, and the distance between the plates was dependent on sample thickness. A frequency sweep test was conducted in the linear viscoelastic regime with a strain of 0.1. Storage modulus (G′) and loss modulus (G″) were evaluated^37^. A uniform thin layer of silicone vacuum grease was applied to the outer circular edges of the shear discs to prevent water loss from the samples^38^.

### In vitro degradation study

The *in vitro* degradation behavior of hydrogels was studied by incubating hydrogel samples (10 mm diameter) in PBS (pH 7.2) in the presence of 0.1% (w/v) NaN_3_ at 37°C. At pre-defined time intervals, the samples were taken out and rinsed with deionized water three times. Then samples were frozen at −20°C overnight and lyophilized for two days. The degradation was characterized by gel fraction (GF) calculated by the following equation: 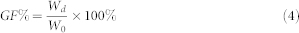
where *W_d_* was the dry weight of the hydrogel sample at certain degradation time point, and *W_0_* was the initial dry weight of the sample.

### Scanning electron microscopy

The cross-section morphologies of the gelatin hydrogels were examined by a Hitachi S-4700 FE-SEM scanning electron microscope (SEM). The accelerating voltage was set to 10 kV with a current 5 mA. The samples were sputter-coated with gold/palladium to a thickness of 10 nm (Hummer 6.2, Anatech LTD) before the observation with SEM.

### Fourier transform infrared spectroscopy

The chemical compositions of the gelatin hydrogels were observed with an attenuated total reflectance-infrared spectroscopy (FTIR-ATR) (Perkin Elmer, MA).

### In vitro cell culture

Gelatin hydrogels were sterilized in 70% ethanol and followed by 3 times of DIH_2_O wash. Bone marrow–derived human mesenchymal stem cells (hMSCs) were provided by TAMU Health Sciences Center. Passage 5 hMSCs were seeded at the density of 5,000 cells/cm^2^ and cultured in alpha-MEM supplemented with 20% FBS, 1% L-glutamine, and 1% penicillin/streptomycin. After 6 and 24 h, samples were fixed and stained with rodamine phalloidin to observe the morphology by Olympus Fluoview FV-1000 confocal fluorescence microscopy (Olympus America, Center Valley, PA). DNA analysis was also performed on 6 and 24 h to determine the cell attachment using PicoGreen assay kit (Life Technologies, Rockville, MD).

## Author Contributions

F.Z. designed the project. Q.X. and F.Z. wrote the main manuscript text. Q.X. prepared figures 2, 5 and 10. K.Y. and Q.X. prepared figures 1, 7 and 8. C.V. and Q.X. prepared figures 3, 4, 6 and supplemental figure 1. Z.Q. and Q.X. prepared the Figure 9. M.F. and F.Z. advised the project. All authors reviewed the manuscript.

## Supplementary Material

Supplementary Information Supplemenatry figure 1

## Figures and Tables

**Table 1:** Calculated parameters used in swelling evaluation of covalent crosslinking in unpurified and purified gelatin hydrogels swollen in PBS at 37°C

Parameters	Unpurified gelatin	Purified gelatin
Equilibrium swelling ratio *Q_m_*	1.6	0.19
Polymer volume fraction *ν*_2*m*_ at swelling equilibrium	0.316	0.796
Crosslinking density 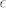 , (×10^−3^), moles/g	1.13	37.3

**Table 2:** Comparison of average storage (G′) and loss moduli (G″) of swelling hydrogels at frequency of 0.42 Hz

		0 h	4 h	24 h	48 h	96 h	7 days
G′ (×10^3^ Pa)	Purified	39.2 ± 13.4	31.2 ± 13.0	14.0 ± 5.93	17.2 ± 6.86	20.3 ± 2.26	16.3 ± 3.51
	Unpurified	16.0 ± 6.10	16.8 ± 5.47	18.1 ± 8.23	19.6 ± 3.04	4.18 ± 0.11	1.76 ± 0.12
G″ (×10^3^ Pa)	Purified	34.5 ± 4.88	20.2 ± 5.83	12.2 ± 4.21	11.7 ± 1.84	13.3 ± 1.18	12.7 ± 1.11
	Unpurified	9.72 ± 4.20	7.94 ± 1.37	5.95 ± 1.43	4.21 ± 1.14	2.45 ± 0.04	1.13 ± 0.05
