# Supplementary material for: Increasing Mechanical Strength of Gelatin Hydrogels by Divalent Metal Ion Removal
Source: Sci Rep. 2014 Apr 16;4:4706. doi: 10.1038/srep04706 (PMC3988488; doi:10.1038/srep04706)

# Increasing Mechanical Strength of Gelatin Hydrogels by Divalent Metal Ion Removal

Qi Xing [a], Keegan Yates [a], Caleb Vogt [a], Zichen Qian [a], Megan C. Frost [b], Feng Zhao *[a]

Supplementary information:

Supplemental Figure 1. EDS of lyophilized unpurified (A) and purified (B) gelatin hydrogels.

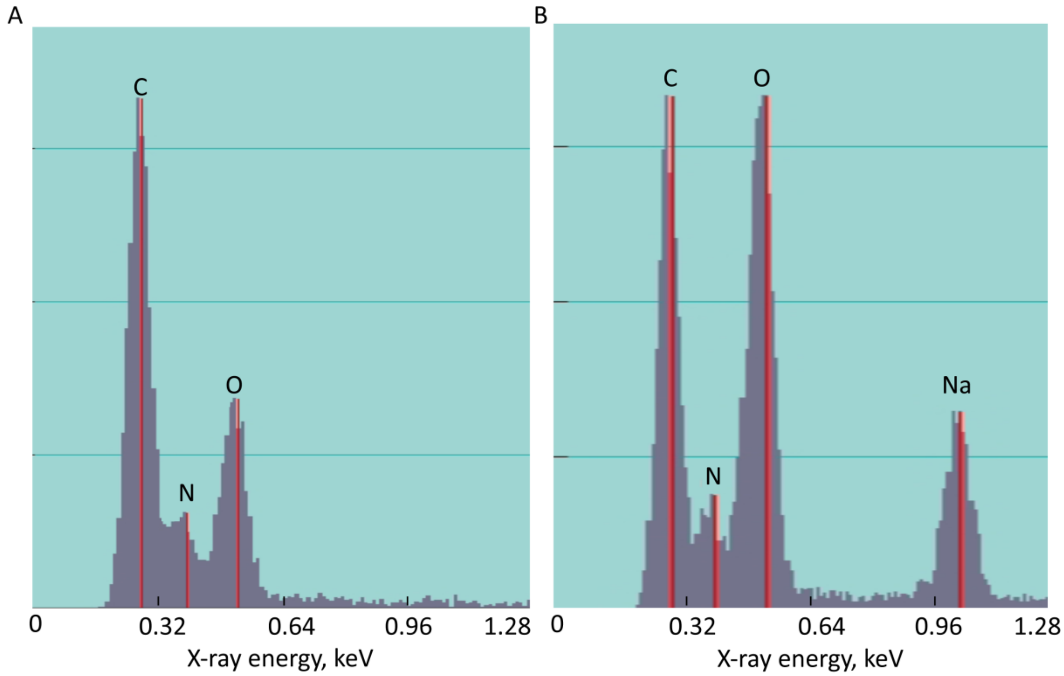

Supplement: Supplementary Information — Supplemenatry figure 1 [file srep04706-s1.pdf]
